# Effects of Chronic Furosemide on Central Neural Hyperactivity and Cochlear Thresholds after Cochlear Trauma in Guinea Pig

**DOI:** 10.3389/fneur.2014.00146

**Published:** 2014-08-08

**Authors:** Wilhelmina H. A. M. Mulders, Courtney McMahen, Donald Robertson

**Affiliations:** ^1^The Auditory Laboratory, School of Anatomy, Physiology and Human Biology, The University of Western Australia, Crawley, WA, Australia

**Keywords:** tinnitus, inferior colliculus, guinea pig, cochleogram, compound action potential

## Abstract

Increased neuronal spontaneous firing rates have been observed throughout the central auditory system after trauma to the cochlea and this hyperactivity is believed to be associated with the phantom perception of tinnitus. Previously, we have shown in an animal model of hearing loss, that an acute injection with furosemide can significantly decrease hyperactivity after cochlear trauma and eliminate behavioral evidence of tinnitus of early onset. However, furosemide also has the potential to affect cochlear thresholds. In this paper, we measured the effects of a chronic (daily injections for 7 days) furosemide treatment on the spontaneous firing rate of inferior colliculus neurons and on cochlear thresholds in order to establish whether a beneficial effect on hyperactivity can be obtained without causing additional hearing loss. Guinea pigs were exposed to a 10–kHz, 124 dB, 2 h acoustic trauma, and after 5 days of recovery, were given daily i.p. injections of 80 mg/kg furosemide or an equivalent amount of saline. The activity of single IC neurons was recorded 24 h following the last injection. The furosemide treatment had no effect on cochlear thresholds compared to saline injections but did result in significant reductions in spontaneous firing rates recorded in inferior colliculus. These results that suggest a long-term beneficial effect of furosemide on hyperactivity after cochlear trauma may be achievable without detrimental effects on hearing, which is important when considering therapeutic potential.

## Introduction

Tinnitus, a phantom auditory perception, is generally thought to be the results of abnormal activity along the central auditory pathways that is often triggered by damage to the auditory receptor, the cochlea (1). One form of abnormal activity observed after cochlear damage is increased spontaneous activity (hyperactivity), which has been shown to be present in cochlear nucleus, inferior colliculus, and auditory cortex (2–6).

Hyperactivity is thought to be the result of an increased central gain following peripheral denervation (7) and available evidence suggests that it is, at least at an early stage, still dependent on the remaining spontaneous drive from cochlear afferents. This has been demonstrated in guinea pigs in which central hyperactivity in inferior colliculus can be eliminated by silencing the cochlea during an early period following acoustic trauma (5), though this effect could not be found at later time points following trauma (8).

If indeed hyperactivity plays a role in the generation of tinnitus, these data suggest that tinnitus of recent onset may be sensitive to treatments affecting the activity of the auditory nerve. This was confirmed in a recent paper in which it was reported that an acute injection with furosemide, at a dose that caused a reduction of the spontaneous activity of the auditory nerve and a reduction of central hyperactivity, also caused an elimination of the early behavioral signs of tinnitus in guinea pigs exposed to cochlear trauma (9). These data, obtained in an animal model, could provide a possible explanation for the fact that VIII nerve transection can decrease tinnitus perception in a proportion of patients (10) and for the fact that furosemide has been shown to suppress the perception of tinnitus in some patients (11–13).

The effects of acute furosemide administration on hyperactivity and tinnitus suggest a therapeutic potential for furosemide in treating early onset tinnitus, although, it is likely that chronic treatment would be needed to achieve ongoing suppression. However, it is also known that furosemide can affect cochlear thresholds, causing hearing loss. Therefore, the present study investigated whether chronic administration of furosemide could decrease hyperactivity without deleterious effects on cochlear thresholds. The effects of a chronic (administered daily for 7 days) furosemide treatment on central hyperactivity and cochlear thresholds were studied in our guinea pig model of hearing loss and hyperactivity after acoustic trauma.

## Materials and Methods

### Animals

Nine adult pigmented guinea pigs of either sex were used. Animals weighed between 290 and 500 g at the time of acoustic trauma. The experimental protocols conformed to the Code of Practice of the National Health and Medical Research Council of Australia, and were approved by the Animal Ethics Committee of The University of Western Australia.

### Recovery surgery for acoustic trauma

Animals received a sub-cutaneous injection of 0.1 ml atropine sulfate (0.6 mg/ml) as pre-medication, followed by an intraperitoneal injection of diazepam (5 mg/kg) and an intramuscular injection of Hypnorm (0.315 mg/ml fentanyl citrate and 10 mg/ml fluanisone; 1 ml/kg). The surgery commenced when there was an absence of the foot withdrawal reflex indicating deep anesthesia. Animals were placed on a heating pad in a sound proof room and positioned in hollow ear bars. Eyes were protected from drying out by application of an eye-gel. A small incision was made behind the left ear and the bulla was opened to visualize the cochlea in order to place an insulated silver wire electrode on the cochlear round window. This enabled recording a compound action potential (CAP) audiogram (14) for the frequency range 4–24 kHz in order to assess the animals’ cochlear sensitivity.

All sound stimuli were presented in a closed sound system through a 1/2″ condenser microphone driven in reverse as a speaker (Bruel and Kjaer, type 4134). Pure tone stimuli were synthesized by a computer equipped with a DIGI 96 soundcard connected to an analog/digital interface (ADI-9 DS, RME Intelligent Audio Solution). Sample rate was 96 kHz. The interface was driven by a custom-made computer program (Neurosound, MI Lloyd), which was also used to collect single neuron data during the final experiments. CAP signals were amplified, filtered (100–3 kHz bandpass) and recorded with a second data acquisition system (Powerlab 4SP, AD Instruments).

When cochlear sensitivity was within the normal range (14), animals received a unilateral acoustic trauma (2 h continuous pure tone of 10 kHz at 124 dB SPL) in the left cochlea while the contralateral ear was blocked with plasticine. Immediately after the acoustic trauma the CAP audiogram was measured again and the incision was sutured. Animals were then monitored hourly until full recovery from anesthesia after which they were returned to the animal housing facility.

### Treatment

Animals were allowed to recover for 5 days after acoustic trauma and were then assigned randomly to either a furosemide group (*n* = 5) or a saline control group (*n* = 4). Intraperitoneal injections of 80 mg/kg furosemide (or an equivalent volume of saline) were administered daily for 7 days (day 5 till day 11 post-trauma). Single neuron recordings in CNIC and CAP threshold measurements (as described above) were taken 1 day after the final furosemide or saline treatment (day 12 post-trauma).

### Surgery for final experiments

Animals received a sub-cutaneous injection with 0.1 ml atropine followed by an intraperitoneal injection of Nembutal (pentobarbitone sodium, 30 mg/kg) and a 0.15 ml intramuscular injection of Hypnorm. Anesthesia was maintained with full Hypnorm doses every hour and half doses of Nembutal every 2 h. When deep anesthesia was obtained as determined by the absence of the foot withdrawal reflex, the areas of incision were shaved and animals were placed on a heating blanket in a sound proof room and artificially ventilated on carbogen (95% O_2_ and 5% CO_2_). The electrocardiogram was continuously monitored and heart rate never increased over pre-paralysis levels at any stage of the experiments. After the animals were mounted in hollow ear bars, the left and right cochleae were exposed and CAP audiograms were recorded on both sides with a silver wire placed on the round window as described for the recovery procedures. Paralysis was then induced with 0.1 ml pancuronium bromide (2 mg/ml intramuscularly).

For extracellular single neuron recordings in the central nucleus of the inferior colliculus (CNIC) a small craniotomy overlying the visual cortex was performed and a glass-insulated tungsten microelectrode (15) was advanced using a stepping motor microdrive along the dorso-ventral axis through the cortex into the CNIC contralateral to the cochlea subjected previously to acoustic trauma. The craniotomy was covered with 5% agar in saline to improve mechanical stability. Electrode placement in the CNIC (about 2.5–3 mm ventral to the cortical surface) was indicated by the presence of strong sound-driven activity with a short latency and a systematic progression from low to high characteristic frequencies (CF) with increasing depth. We have previously confirmed histologically that these response properties correlate with location of the electrode in the CNIC (16). When a single unit was isolated its CF and threshold at CF were determined audio-visually and depth from the cortical surface was recorded. Spontaneous firing rate was measured for a period of 10 s.

### Data analysis

Hearing loss can result in changes to the tuning curves of neurons, so that determination of CF based on the lowest threshold of tuning curves in the damaged region can be erroneous (17). We therefore calculated a nominal CF for all neurons collected in this study based on the recorded unit’s electrode depth into the CNIC (from cortical surface) during post-experimental analysis [See also Ref. (5, 16)]. The highly structured tonotopic map of the IC allows for CF to be estimated using depth measurements. In each animal, we constructed a plot of audio-visually determined CFs from all neurons in regions that were unaffected by the acoustic trauma against their depth into the CNIC. A second-order polynomial function was then fitted to these plots and the formula of the fitted curve was then used to transform depth into nominal CF. Nominal CF will be used in the remainder of the manuscript.

To identify statistically significant differences in spontaneous firing rates between chronic intraperitoneal injections with furosemide or saline, the Mann–Whitney test was used when comparing all neurons in each group with each other and the Kruskall–Wallis test and Dunn’s multiple comparison post-test when comparing the neurons sorted according to CF range. Statistical analysis of CAP threshold changes at each frequency was performed using a one-way ANOVA.

## Results

### Effects of chronic furosemide on peripheral thresholds

**Figure 1:**
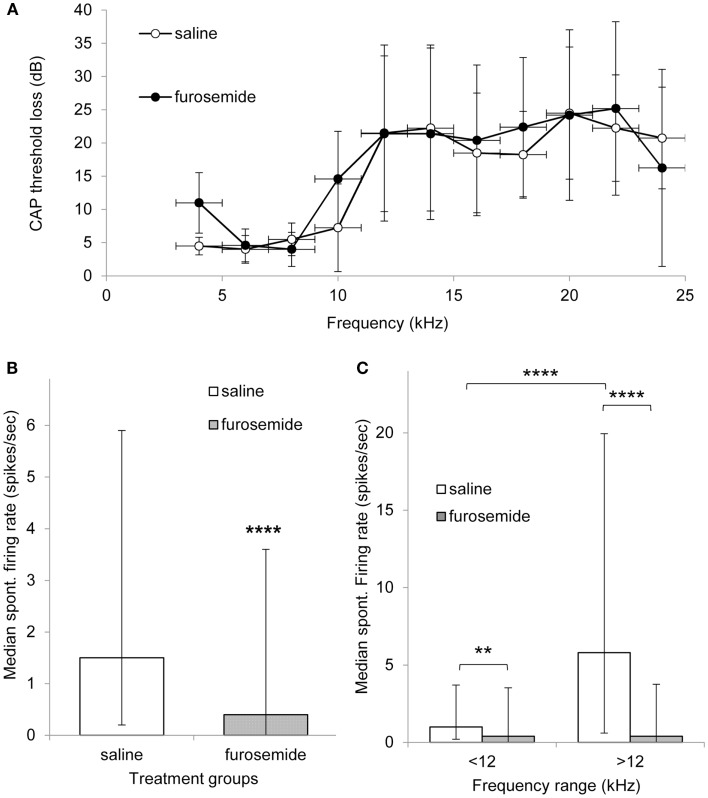
**(A)** Mean threshold loss (mean ± SEM) at multiple frequencies after recovery from acoustic trauma (day 12) from animals that were treated with saline (open circles, *n* = 4) of furosemide (black circles, *n* = 5). **(B)** Histogram showing median spontaneous firing rate in CNIC in saline (362 neurons from four animals, white bar) and furosemide treated groups (398 neurons from five animals, black bar) 12 days after acoustic trauma. Median shown with 25 and 75% percentile. **(C)** Histogram showing median spontaneous firing rate of CNIC neurons in high and low frequency regions in saline treated animals (white bars) and furosemide treated animals (black bars) on day 12 after acoustic trauma. Bars show median with 25 and 75% percentile **p < 0.01; ****p < 0.0001.

Figure 1A shows the average CAP threshold loss in the exposed ear of both saline and furosemide treated animals on day 12 after the acoustic trauma. Both groups of animals showed CAP threshold losses at frequencies >10 kHz in line with data published previously using the same animal model (5, 16). Furthermore, there were no statistically significant differences between the groups at any frequency, including the lower frequencies that were unaffected by the acoustic trauma. These results show that the chronic administration of furosemide did not have additional lasting effects on the peripheral thresholds.

### Effects of chronic furosemide on central neural activity

In total, data were obtained from 362 neurons in the CNIC of saline treated animals and 398 neurons from the CNIC of furosemide treated animals. Spontaneous firing rates in saline treated animals varied between 0 and 77.6 spikes/s with a mean spontaneous firing rate of 6.5 ± 0.65 (median 1.5) spikes/s and in furosemide treated animals between 0 and 52.5 spikes/s with an average rate of 3.45 ± 0.36 spikes/s (median 0.4) (Figure 1B). The spontaneous firing rate in furosemide treated animals was significantly reduced compared to saline treated animals (*p* < 0.0001).

In order to investigate whether furosemide was equally affecting the spontaneous firing rate of all neurons throughout the tonotopic map of the IC, neurons were also sorted according to CF. The spontaneous firing rates in saline and furosemide treated animals were compared for neurons with a CF < 12 kHz (non-hearing loss region) and a CF > 12 kHz (hearing loss region). Results from this analysis are shown in Figure 1C. In agreement with previously published data that showed a strong correlation between the frequency regions in the CNIC showing increased spontaneous firing and the peripheral frequency regions of hearing loss (16), Figure 1C shows statistically elevated levels of spontaneous firing rate in the CF region >12 kHz compared to the CF region <12 kHz in saline treated animals (*p* < 0.0001). Statistical analysis showed a significant decrease after furosemide in both CF groups (*p* < 0.01 for CFs <12 kHz and *p* < 0.0001 for CFs >12 kHz). The largest effect of furosemide was seen in the frequency region that showed the most prominent hyperactivity.

## Discussion

The results presented in this paper show that 1 day after a 7-day course of furosemide central hyperactivity in inferior colliculus is reduced without effects on peripheral auditory thresholds. These results are in agreement with the results of single acute furosemide injections, which also result in suppression of hyperactivity and they show that chronic administration can have beneficial effects centrally without causing further hearing loss. Further experiments are required to investigate the duration of the benefit from repeated administration and whether this is accompanied by a concomitant reduction of tinnitus.

Importantly, chronic administration of furosemide did not affect the magnitude of the hearing loss following acoustic trauma compared to saline injections. This indicates that furosemide did not have long-lasting effects on cochlear thresholds, even though studies do suggest a possible short-term effect on sound-evoked responses in the cochlea (18–20). Furosemide, a loop-diuretic, is known to decrease the endocochlear potential in the cochlea via an effect on the Na-K-2Cl co-transporter in the stria vascularis (21). This can lead to temporary decreases in the spontaneous firing of the auditory nerve (20, 22), increases in the thresholds of single auditory nerve fibers, and a reduction in the CAP amplitude of the auditory nerve (18–20). However, the latter studies did report that the effects of the furosemide doses used (100 mg/kg in chinchilla, or i.v. infusion in cats at 10 mg/ml) were reversible and significantly attenuated within an hour of treatment (18–20). Furthermore, Sewell (19) also showed that the spontaneous firing of primary afferent neurons was more susceptible to furosemide effects than was auditory sensitivity. This is also in agreement with our previous study showing initial increases in neuronal thresholds in IC after intracochlear perfusion of furosemide, which recovered fully after 1 h (9). The dose of furosemide used in this study may therefore have decreased the sound-evoked activity for a brief period after the injection, but there was no long-lasting effect on peripheral thresholds as the CAP audiogram data indicate.

Overall spontaneous firing rates were significantly reduced after the chronic treatment with furosemide as compared to saline. This is the same effect as observed after acute administration of furosemide at the same dosage (9). In addition, this reduction is most striking in the tonotopic regions >12 kHz, although it was significantly suppressed in the frequency region <12 kHz as well. It is noteworthy that this effect of furosemide was seen 24 h after the last injection, whereas our previous study investigated effects only immediately (within 4 h) after a single acute injection. The hyperactivity is most pronounced in the frequency region >12 kHz, which confirms earlier observations that there is a strong correlation between the frequency regions showing hyperactivity and the frequency extent of hearing loss (2, 16, 23, 24). The reduction in hyperactivity looks similar to that described previously following complete cochlear ablation, cochlear cooling, and acute administration of furosemide (5, 9), providing further evidence that hyperactivity can be modulated by manipulations of auditory nerve activity.

What is the cause of the reduction in spontaneous firing rates in IC? Firstly, the level of hyperactivity is known to be affected by the magnitude of hearing loss (16); however, no difference in hearing loss was apparent between the saline and furosemide treated animals. In addition, since furosemide is thought to increase cochlear thresholds (18–20), it would be expected to enhance the hearing loss and thereby increasing the central spontaneous firing rates. Secondly, the reduction in hyperactivity could be a direct result of the reduced spontaneous activity in the auditory nerve caused by furosemide nerve (9, 20, 22). As we have shown previously, the dose of furosemide used in this study acutely decreases the spontaneous firing rate in the cochlear nerve and also reduces the hyperactivity in the IC (9). The 7-day course of furosemide may have caused a long-term decrease of the auditory nerve spontaneous firing rate without affecting the thresholds, which is very well possible since the spontaneous firing rate is more sensitive to suppression of the endocochlear potential than cochlear thresholds (22).

In view of the fact of our previous study showing that acute administration of furosemide lowers the hyperactivity in IC and eliminates the behavioral signs of tinnitus (9), the present results suggest that chronic furosemide may have longer-lasting beneficial effects on tinnitus and can be administered without detrimental effects on hearing thresholds. This is an important observation when considering furosemide as a treatment for early onset tinnitus. However, we do note that in the time window of the present study animals are unlikely to have developed tinnitus yet since we observed this between 4 and 6 weeks after cochlear trauma (9, 24). It is therefore necessary to investigate the effect of chronic furosemide at later time points as well and establish whether it is accompanied by a longer-lasting effect on tinnitus. Finally, it is also of interest to research whether an early reduction of the hyperactivity could prevent the establishment of behavioral tinnitus, which would further support a role for the hyperactivity in the generation of tinnitus.

## Conflict of Interest Statement

The authors declare that the research was conducted in the absence of any commercial or financial relationships that could be construed as a potential conflict of interest.
